# Oxidative Stress and DNA Lesion Reduction of a Polyphenolic Enriched Extract of *Thymus marschallianus* Willd. in Endothelial Vascular Cells Exposed to Hyperglycemia

**DOI:** 10.3390/plants10122810

**Published:** 2021-12-18

**Authors:** Irina Ielciu, Gabriela Adriana Filip, Ilioara Oniga, Neli-Kinga Olah, Ioana Bâldea, Diana Olteanu, Ramona Flavia Burtescu, Violeta Turcuș, Alexandra C. Sevastre-Berghian, Daniela Benedec, Daniela Hanganu

**Affiliations:** 1 Department of Pharmaceutical Botany, Faculty of Pharmacy, “Iuliu Haţieganu” University of Medicine and Pharmacy, 400010 Cluj-Napoca, Romania; irina.ielciu@umfcluj.ro; 2 Department of Physiology, Faculty of Medicine, “Iuliu Haţieganu” University of Medicine and Pharmacy, 400006 Cluj-Napoca, Romania; ioana.baldea@umfcluj.ro (I.B.); ariana_di@yahoo.com (D.O.); berghian.alexandra@umfcluj.ro (A.C.S.-B.); 3 Department of Pharmacognosy, Faculty of Pharmacy, “Iuliu Haţieganu” University of Medicine and Pharmacy, 400010 Cluj-Napoca, Romania; dbenedec@umfcluj.ro (D.B.); dhanganu@umfcluj.ro (D.H.); 4 Department of Pharmaceutical Chemistry, Faculty of Pharmacy, “Vasile Goldiş” Western University of Arad, 310414 Arad, Romania; neli.olah@plantextrakt.ro; 5 PlantExtrakt Ltd., Rădaia, 407059 Cluj-Napoca, Romania; ramona.burtescu@plantextrakt.ro; 6 Department of Botany, Faculty of Medicine, “Vasile Goldiş” Western University of Arad, 310414 Arad, Romania; violeta_buruiana@yahoo.com

**Keywords:** *T. marschallianus*, polyphenolic enriched extracts, oxidative stress, NF-ĸB, HIF-1, γ-H_2_AX

## Abstract

The present study aimed to compare two polyphenolic-enriched extracts obtained from the *Thymus marschallianus* Willd. (Lamiaceae) species, harvested from culture (TMCE in doses of 0.66 μg GAE/mL and 0.066 μg GAE/mL) and from spontaneous flora (TMSE in doses of 0.94 μg GAE/mL and 0.094 μg GAE/mL) by assessing their biological effects on human umbilical vein endothelial cells (HUVECs) exposed to normoglycemic (137 mmol/L glucose) and hyperglycemic conditions (200 mmol/L glucose). Extracts were obtained by solid phase extraction (SPE) and analyzed by chromatographical (HPLC-DAD) and spectrophotometrical methods. Their effects on hyperglycemia were evaluated by the quantification of oxidative stress and NF-ĸB, pNF-ĸB, HIF-1α, and γ-H_2_AX expressions. The HPLC-DAD analysis highlighted significant amounts of rosmarinic acid (ranging between 0.18 and 1.81 mg/g dry extract), luteolin (ranging between 2.04 and 17.71 mg/g dry extract), kaempferol (ranging between 1.85 and 7.39 mg/g dry extract), and apigenin (ranging between 4.97 and 65.67 mg/g dry extract). Exposure to hyperglycemia induced oxidative stress and the activation of NF-ĸ increased the expression of HIF-1α and produced DNA lesions. The polyphenolic-enriched extracts proved a significant reduction of oxidative stress and γ-H_2_AX formation and improved the expression of HIF-1α, suggesting their protective role on endothelial cells in hyperglycemia. The tested extracts reduced the total NF-ĸB expression and diminished its activation in hyperglycemic conditions. The obtained results bring evidence for the use of the polyphenolic-enriched extracts of *T*. *marschallianus* as adjuvants in hyperglycemia.

## 1. Introduction

Diabetes mellitus (DM) is a group of pathological alterations characterized by hyperglycemia in the absence of treatment [1], which may come from insulin resistance, from inadequate insulin secretion or from disturbances in the metabolism of carbohydrates, fats, and proteins [1,2,3]. The classification of diabetes includes two types of diseases: type 1 (T1DM), an autoimmune condition characterized by the destruction of pancreatic beta cells, and type 2 (T2DM), a more common pathology characterized by impaired glucose regulation as a consequence of dysfunctional beta pancreatic cells and insulin resistance [1,3,4,5]. DM is found in all regions of the world, including low-income and rural areas [1,3]. The number of people suffering from DM is continuously increasing. Thus, WHO estimated in 2014 that 422 million adults were suffering from DM and the prevalence in adults almost doubled from 1980 to 2014 (from 4.7% to 8.5%), with more than 1.1 million children and adolescents suffering from DM. It is estimated that 1 in 11 people are living with diabetes and more than 700 million adults worldwide will have diabetes by 2045 [1]. The most affected age category remains the elderly, with a prevalence and mortality higher than in younger people [6].

Diabetes is a chronic disease with numerous complications from macrovascular and microvascular damage [2]. Microvascular complications affect small vessels from the kidneys, eyes, and nerves and include nephropathy, retinopathy, and neuropathy, producing renal failure, blindness, impotence, and diabetic foot, which can end in infections and amputations. Macrovascular damage can induce cardiac disorders, such as stroke, heart attack, and insufficient blood flow in the legs [2,7]. The prevalence of complications is higher in elderly people and vascular abnormalities are frequently associated in their case [6].

In the pathophysiology of DM, oxidative stress plays an important role, especially in the development of vascular complications. In fact, hyperglycemia can increase the reactive oxygen species (ROS) production through different mechanisms concerning enzymes of the mitochondrial respiratory chain or xanthine oxidases, lipoxygenases, cyclooxygenases, nitric oxide synthases, and peroxidases activities [8]. A reduction in enzyme activity makes the tissues susceptible to the effects of oxidative stress and consequently to the complications of diabetes [9]. Additionally, hyperglycemia induces advanced glycation end products (AGEs) formation which can trigger the pro-inflammatory response via the activation of NF-κB [10].

An important mechanism involved in the insulin resistance in T2DM is related to the nuclear transcription factor (NF)-ĸB, a key factor involved in the cellular response to different stimuli such as stress, cytokines, free radicals, UV irradiation, and microorganisms [11]. In hyperglycemia, NF-kB expression increases and the cytokines, TGF-β, chemokines, and vascular cell adhesion molecules (VCAMs) are released. The upregulation of TNF-α, IL1β, IL6, CD36, and MCP-1 induces endothelial cell inflammation and apoptosis. Moreover, over-activated NF-κB triggers the abnormal transcription of DNA and various genes involved in vascular complications. The over-activity of NF-κB also leads to the altered gene expression of vascular endothelial growth factor (VEGF), platelet-derived growth factor (PDGF), endothelin-1 (ET-1), activated protein C (APC), and transforming growth factor-β (TGF-β), which ends in vascular cell damage and retinal angiogenesis [11,12].

Other molecules involved in the pathogenesis of DM are HIF-1 and γ-H_2_AX [13,14]. HIF-1α is the transcription factor essential for the adaptive responses of cells to hypoxia. In exposure to hypoxia, HIF-1α upregulates a series of genes implicated in angiogenesis, glycolytic energy metabolism, wound healing, and cell proliferation and survival. At the same time, the inability of cells to respond to hypoxia can increase the risk of complications in DM [13]. γ-H_2_AX is a marker of the formation of double-strand breaks as a response to exposure to DNA damaging factors and has been used for drug development and clinical studies on diabetes [15].

The effect of natural polyphenols in DM management and prevention is important, as they have been proven to have abilities such as inhibiting carbohydrate digestion (by α-amylase and α-glucosidase), inhibiting glucose absorption, stimulating insulin secretion, and protecting pancreatic β-cells against glucotoxicity [16,17,18,19,20,21]. All these mechanisms of action are directly linked with their main biological activity, as most natural polyphenols have important antioxidant properties [21].

*Thymus marschallianus* Willd., Hungarian thyme, belonging to the genus *Thymus* and to the family Lamiaceae, is a Eurasian species that has not been sufficiently studied in the scientific literature, due to the fact that for many years it has been considered to be a synonym of another taxa, *T. pannonicus* All., which has been proven to be false, as the two represent completely different taxa [22,23,24]. The differences between them are related to their botanical characters and concern the presence of trichomes, especially for the *T. pannonicus* species, which has long multi-cellular trichomes, while *T*. *marschallianus* has no trichomes. Differences between the two taxa may be found also in the chemical composition of essential oils [22]. The chemical composition of *T*. *marschallianus* is quite similar to other *Thymus* species, as it was proved to contain both essential oils and polyphenols [22,25,26,27]. Among the investigated pharmacological properties for this species, the antioxidant [26,27], antimicrobial [22,26,27], and antidiabetic potential [14] appear to be the most important.

SPE is a selective and rapid method of obtaining extracts enriched in bioactive compounds, including polyphenols, using low processing temperatures and low amounts of solvents while at the same time minimizing the effects of the oxidation reactions [28]. By using this method, the obtained extracts may show effective biological effects due to a particular polyphenolic profile in which specific compounds are present in different concentrations.

Taking all this into consideration, the present study aims to evaluate the effect on the oxidative stress, transcription factors, and DNA lesions of polyphenolic-enriched extracts obtained from *T*. *marschallianus* on endothelial vascular cells exposed to hyperglycemia. The two tested samples consisted of two different extracts obtained from this species, collected from different environments, both from the spontaneous flora (TMSE) and from culture (TMCE), and were chosen to compare the influence of external conditions (pedoclimatic, agrotechnical) on the polyphenolic profile and on the biological activities. The originality and novelty of the study is related to the fact that scientific data on the antidiabetic activity of this species are scarce, especially those related to the mechanism of action of the polyphenolic compounds in DM. Moreover, the comparison between these different types of samples was performed in order to establish if the quality of the vegetal material obtained from culture presents a similar chemical composition and biological activities as that obtained from the spontaneous flora, therefore proving its potential to help increase the use of this material instead of the limited material that may be obtained from spontaneous flora. In this way, the potential of introducing the species in different spreading areas appears to be an important consideration regarding this species. At the same time, the research tries to bring scientific arguments for the antidiabetic potential, proving that the species has similar therapeutic potential to numerous other species of the genus.

## 2. Results

### 2.1. HPLC Quantification of Polyphenolic Compounds and Spectral Quantification of Total Polyphenols (TPC), Total Phenolic Acids (TPA), and Flavonoids Content (TFC)

The two tested polyphenolic-enriched extracts of *T*. *marschallianus* collected from spontaneous flora (TMSE) and from culture (TMCE) were analyzed by a HPLC-DAD method and the results were compared with the corresponding extracts before enrichment in polyphenols (TMS for the *T*. *marschallianus* sample collected from spontaneous flora; TMC for the *T*. *marschallianus* sample collected from culture). A comparison between extracts before and after enrichment was performed in order to highlight the differences between the two types of samples and to reveal the significantly increased amounts of compounds that were obtained in the polyphenolic-enriched extracts.

The choice of testing these two samples represents one of the objectives of the study and, at the same time, is an element of novelty. In fact, wild flora represents a limited resource, not only because of the intervention of external factors on different species, but also due to the limited distribution area. For this reason, the *T*. *marschallianus* species was introduced into the culture [29], obtaining a quality plant material with high productivity. Starting from the advantages of the agricultural technology, which imply obtaining a quality vegetal material, introduction into culture appears necessary and, with it, it becomes essential to test and compare samples obtained from spontaneous flora and from culture, both from the point of view of the chemical composition and of their biological activities. In this context, the comparative study is logical and brings novelty and originality to the present research.

The identification of different polyphenols was performed using the retention times and the UV-Vis spectra of separated compounds and standards. The position of the maximal absorbance from the obtained UV-Vis spectra was used for identification. All separated compounds having a specific shape of spectra, meaning a maximum from 230 to 300 nm and a more specific range between 320 and 335 nm and between 335 and 370 nm, respectively, were considered to belong to the class of polyphenols, to the class of phenolic acids if the specific maximum of absorption was at 320–335 nm and to flavonoids if the specific absorbance maximum was at 335–370 nm. The results of the phytochemical analysis of the two tested samples are presented in Table 1 and Table 2 and are expressed as milligrams of caffeic acid equivalents (CAE) for total phenolic acids (TPA), milligrams of rutoside equivalents (RE) for total flavonoids (TFC), milligrams of gallic acid equivalents (GAE) for total polyphenols (TPC), and milligrams of rosmarinic acid, luteolin, kaempferol, and apigenin per gram of dry extract.

**Figure 1:**
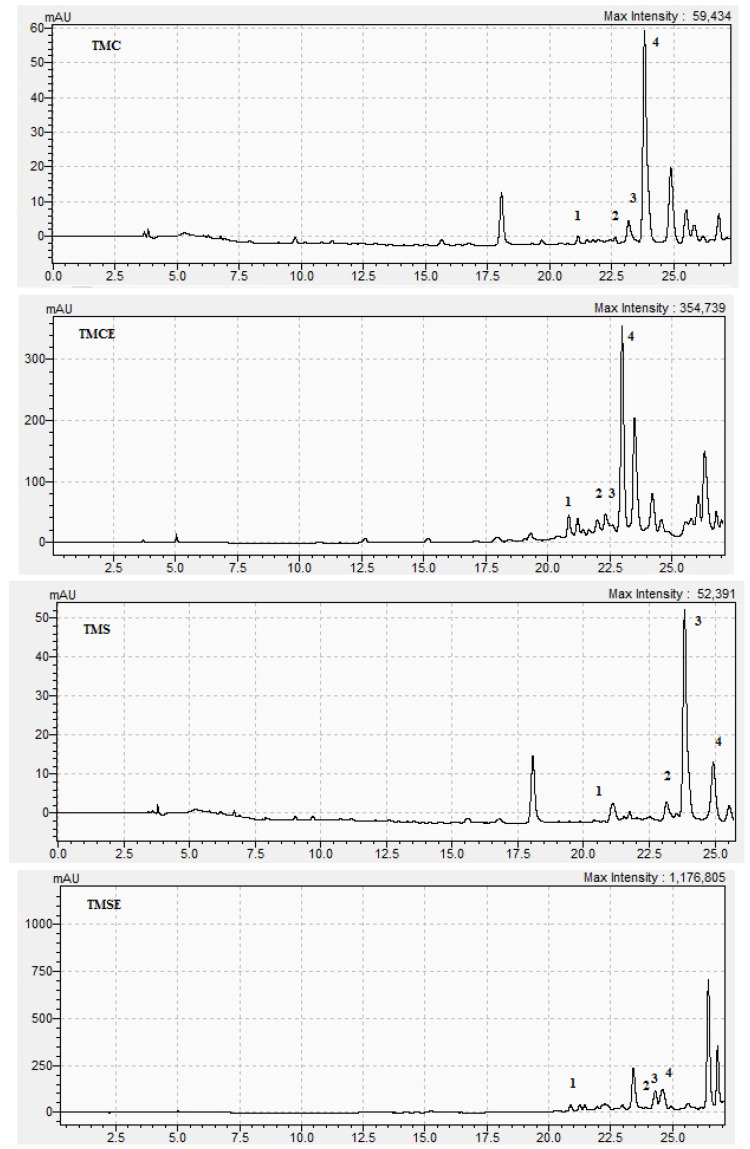
HPLC-DAD chromatograms at 254 nm for 1 = luteolin, 2 = kaempferol, 3 = apigenin, and 4 = rosmarinic acid.

It can be clearly observed that, when comparing the composition of extracts, the polyphenolic-enriched extracts for both samples had significant differences. There are higher amounts of the TPC, TFC, and the individual compound (rosmarinic acid, luteolin, kaempferol and apigenin) in the enriched extracts (Figure 1). Polyphenolic-enriched extracts were further used for biological assays.

### 2.2. Cell Viability

**Figure 2:**
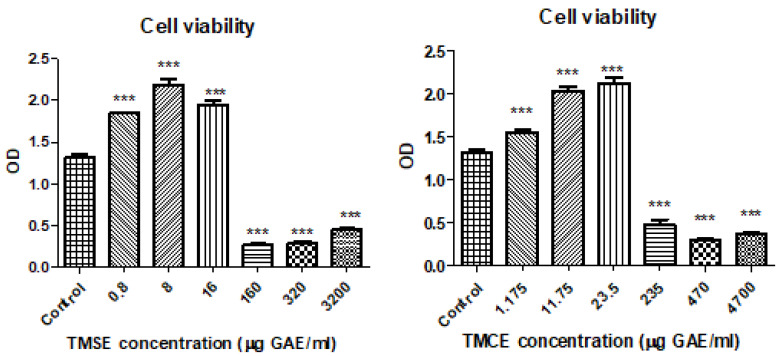
Cell viability of HUVECs treated with TMSE and TMCE in different concentrations compared to the control (untreated cells). HUVECs were exposed for 24 h to the TMSE and TMCE extracts in concentrations ranging between 0.8 and 3200 μg GAE/mL and between 1.175 and 4700 μg GAE/mL, respectively, compared to the control (untreated cells). Data are presented as a mean of OD ± SD, n = 3 for each sample. *** *p* < 0.001 vs. control.

HUVECs exposed to normoglycemic and hyperglycemic medium were used in order to assess the toxicity of two tested samples. The exposure of HUVECs to TMSE and TMCE extracts did not lead to any significant changes in cell viability at low concentrations compared to the controls. Moreover, low levels of polyphenols increased the viability significantly in a dose dependent manner up to 16 μg GAE/mL for TMSE and 23.5 μg GAE/mL for TMCE (Figure 2). For the two extracts, the high doses reduced the cell viability (at doses above 160 µg GAE/mL for TMSE and 235 µg GAE/mL for TMCE). Based on the viability test, the concentrations chosen for further experiments were for TMSE 0.66 μg GAE/mL and 0.066 μg GAE/mL and for TMCE 0.94 μg GAE/mL and 0.094 μg GAE/mL.

### 2.3. Influence of T. marschallianus on Oxidative Stress

**Figure 3:**
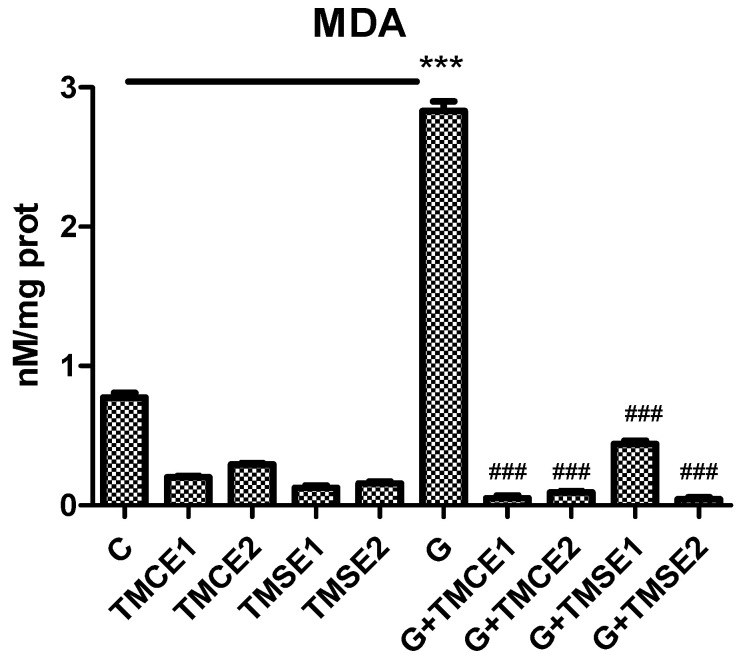
Malondialdehyde (MDA) levels in HUVECs after exposure to hyperglycemia and normoglycemia and after treatment with two different doses of TMCE and TMSE. Exposure to hyperglycemia (G) significantly increased MDA levels in cell lysates (*p* < 0.001) compared to the control while TMCE and TMSE in both concentrations reduced MDA formation (*p* < 0.001). Statistical significance of the difference between control groups and the ones treated with samples was evaluated with two-way ANOVA and Bonferroni post-hoc test. *** *p* < 0.001 vs. control cells and ^###^ *p* < 0.001 vs. cells exposed to hyperglycemia.

Malondialdehyde (MDA) levels were quantified in normoglycemic and also in hyperglycemic conditions in order to establish the effect of the two samples on the redox imbalance. It can be clearly observed that hyperglycemia caused the oxidation of the membrane lipids of HUVECs, as displayed by the MDA levels in the cells exposed to hyperglycemic medium (G vs. C, *p* < 0.001). The two *T*. *marschallianus* extracts, in both concentrations, significantly reduced MDA levels in the cells exposed to hyperglycemia without any difference between the groups (Figure 3).

### 2.4. Influence of T. marschallianus on Transcription Factors in HUVECs

**Figure 4:**
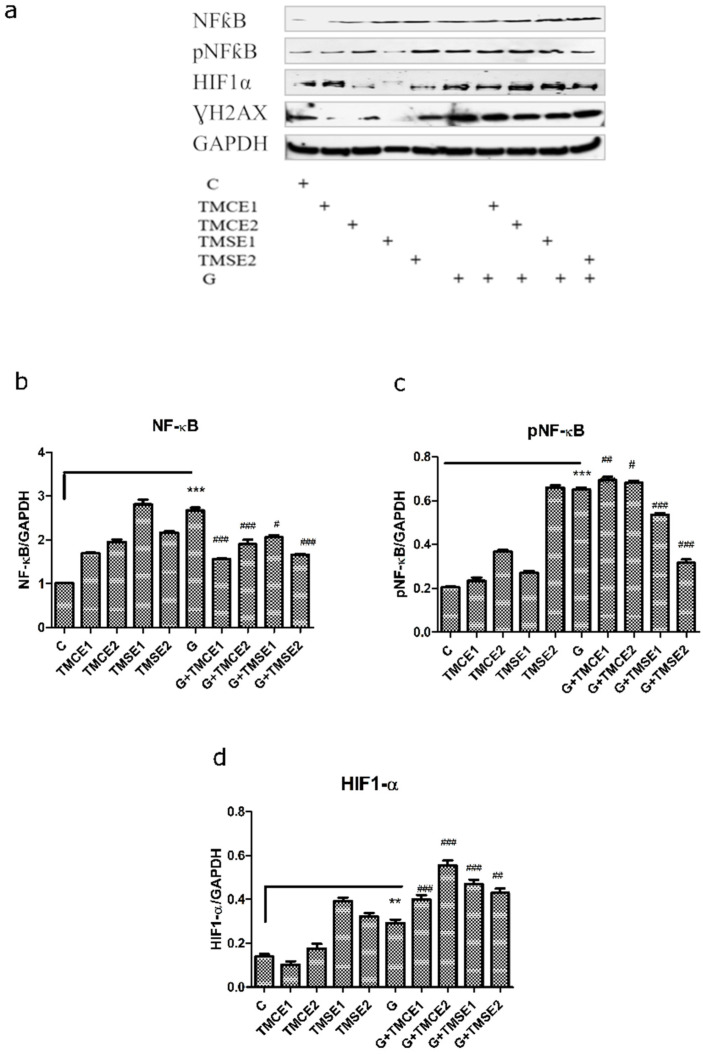
NF-ĸB, phosphorylated (p)NF-ĸB and HIF1-α expressions in HUVECs after exposure to hyperglycemia and normoglycemia and treatment with two doses of *Thymus marschallianus* extracts. Image analysis of WB bands was done by densitometry in upper panels (**a**) and results were normalized to GAPDH in lower panels (**b**–**d**). Exposure to hyperglycemia (G) increased the total NF-kB expression and its activation and enhanced the HIF1-α valued compared to the control cells. The treatment with the two extracts diminished the total NF-kB expression and reduced the active form of NF-kB, especially TMSE. Both extracts improved HIF1-α expression in hyperglycemic medium while TMCE increased the activation of NF-kB. The statistical significance between the treated cells and control group was assessed with two-way ANOVA followed by the Bonferroni post-hoc test. Each bar represents mean ± standard deviation (n = 3); ** *p* < 0.01 and *** *p* < 0.001 vs. control (untreated cells); ^#^ *p* < 0.05, ^##^ *p* < 0.01, and ^###^ *p* < 0.001 vs. cells exposed to hyperglycemia.

The quantification of the effect of two doses of the extracts on the NF-ĸB pathway was performed by the Western blot analysis of the constitutive and phosphorylated forms in lysates obtained from treated and untreated HUVECs. NF-kB protein levels increased in the cells exposed to hyperglycemia compared to the control cells exposed to normoglycemia (*p* < 0.001). Both doses of two extracts reduced the total NF-kB protein expression in HUVECs incubated in hyperglycemic conditions (*p* < 0.001). The phosphorylated form of NF-kB enhanced after exposure to hyperglycemia or hyperglycemia associated with both doses of TMCE extract (*p* < 0.01 and *p* < 0.05), suggesting the stimulatory effect of TMCE extract on NF-kB activation. The NF-kB phosphorylated form represents a key factor involved in the transcription of genetic information from the nucleus and in the activation of enzymes with a role in inflammation and cell proliferation. NF-kB activation decreased after the treatment of cells with both doses of TMSE (*p* < 0.001) (Figure 4). In cells exposed to hyperglycemia, HIF1-α expression enhanced significantly (*p* < 0.01), this behavior being amplified by the association of the two extracts (*p* < 0.001, *p* < 0.01). This effect of TMCE and TMSE extracts on HIF1-α expression, in cells incubated in hyperglycemic medium, suggests the beneficial role of polyphenols in angiogenesis and vascular protection.

### 2.5. Influence of T. marschallianus on DNA Lesions in HUVECs

**Figure 5:**
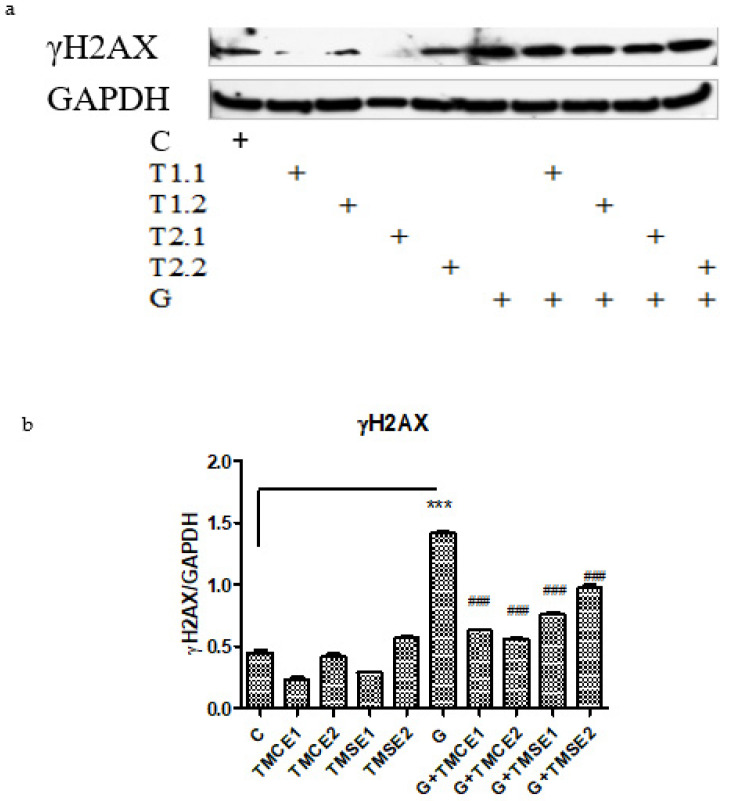
HIF-1α and γ-H_2_AX expressions in HUVECs after exposure to hyperglycemia and normoglycemia and after treatment with *T*. *marschallianus* extracts. Image analysis of WB bands was done by densitometry in upper panels (**a**) and results were normalized to GAPDH in lower panels (**b**). The exposure to hyperglycemic medium induced DNA lesions while the two extracts exerted the protection on DNA damage. The statistical significance between the treated cells and control group was assessed with two-way ANOVA followed by the Bonferroni post-hoc test. Each bar represents mean ± standard deviation (n = 3); *** *p* < 0.001 vs. control (untreated cells); ^###^ *p* < 0.001 vs. cells exposed to hyperglycemia.

Compared to the control cells, in hyperglycemic conditions, γ-H_2_AX expression increased significantly, suggesting the role of hyperglycemia in the induction of DNA lesions. After exposure to *T*. *marschallianus* extracts, γ-H_2_AX levels decreased significantly independent of the dose or the type of extract used (Figure 5), indicating a vascular protection property.

## 3. Discussion

In order to find new natural remedies in diabetes, the present study investigated the biological activities of two polyphenolic-enriched *T*. *marschallianus* extracts, prepared from samples harvested from culture (TMCE) and spontaneous (TMSE) flora, on human umbilical vein endothelial cells (HUVECs) exposed to hyperglycemia. The results showed that hyperglycemia induced oxidative stress and the activation of NF-kB, increased the expression of HIF1-α, and triggered DNA lesions. These two extracts proved a significant antioxidant activity, inhibited the γ-H_2_AX formation, and improved the expression of HIF1-α, suggesting their protective role on endothelial cells exposed to hyperglycemia. Moreover, TMSE reduced the total protein NF-kB expression and diminished its activation in hyperglycemic conditions, exerting a beneficial activity on inflammation and apoptosis in endothelial vascular cells. Previous studies performed on animal models with diabetes have demonstrated that the two extracts, TMS and TMC, diminished blood glucose levels and lipid peroxidation and enhanced the antioxidant capacity in the frontal lobe. TMS improved the central locomotion of rats, increased phospho-NFkB p65, and diminished the methyl CpG binding protein (MECP) 2 expressions in the hippocampus [14]. In this study, the same extracts, but enriched in polyphenols, were used for in vitro experiments on endothelial cells exposed to hyperglycemia.

The phytochemical analysis of the extracts helped to identify and quantify four individual polyphenols, belonging to the class of phenolic acids (rosmarinic acid) and flavonoids (luteolin, kaempferol, apigenin). These compounds were previously identified in the composition of the species by our group of researchers [14,22] and by other authors [27]. The comparative results of the initial extracts and the concentrated ones showed that the polyphenols and flavonoids content increased by SPE concentration with 80.8% and 134.5%, respectively, from the sample collected from culture and with 34.7% and 94.1%, respectively, in the case of the sample harvested from the spontaneous flora. The results obtained in the quantitative analysis clearly showed higher values for TFC in both samples. In the DAD determination, it could be observed that the phenolic acids were found in higher amounts in the sample collected from the spontaneous flora, but the flavonoids were found in higher amounts in the sample obtained from culture. Generally, the sample obtained from culture was richer in polyphenols compared to the sample collected from the spontaneous flora. The enrichment in polyphenols of the extract obtained from the sample collected from culture was higher than the one of the extracts obtained from the species harvested from the spontaneous flora. The TMCE sample had a very high enrichment in apigenin at 1220.8% (13.2 times higher), in luteolin at 770.3% (8.7 times higher), and in kaempferol at 300% (4 times higher), and was less enhanced in rosmarinic acid at 64.7% (2.34 times higher), while the TMSE was enriched more in apigenin at 123% (2.66 times higher) and in kaempferol at 106.8% (2.06 times higher), and was less enriched in luteolin at 36.4% (1.31 times higher) and in rosmarinic acid at 37.5% (1.37 times higher). The enrichment conditions therefore favored the increase in concentrations of flavonoids more than in any other phenolic acids. Thus, a richer phytochemical profile of the sample obtained from culture, compared to the one obtained from the wild flora, can also be observed. SPE concentration conditions brought therefore a higher concentration in aglycons and a lower concentration in glycosides.

Moreover, based on the background of the compounds identified in the two extracts and their role in glucose metabolism and glucose regulation, our research aimed to evaluate the oxidative stress associated with hyperglycemia and related signaling pathways. Studies have demonstrated that apigenin improved the glucose metabolism in a mouse fed a high-fat diet [30] and reduced obesity and obesity-related insulin resistance as well as exerted anti-inflammatory, antioxidant, and hypoglycaemic effects by the inhibition of endoplasmic reticulum stress (ERS) [31]. Rosmarinic acid has been shown to have an insulin-like effects in target cells and increase the hepatocyte glucose consumption and glycolytic rate. Additionally, rosmarinic acid protected the hepatocytes against reactive oxygen species toxicity and prevented cell death by apoptosis, in both in vitro and in vivo studies [32].

Based on these data, it seems that targeting oxidative stress may represent a strategy to follow for the prevention and control of DM [33]. In order to prove the antidiabetic potential of TMSE and TMCE, HUVECs were exposed to a hyperglycemic medium and the results showed that hyperglycemia induced oxidative stress, an effect which was diminished by the administration of the two extracts.

Moreover, oxidative stress is strongly correlated with a high incidence of DM and with the development of complications. Both the insulin secretion and the activity of insulin are deficient during DM and it seems to be due to oxidative stress [34,35]. Hyperglycemia is directly related to the high levels of free radicals resulting from the non-enzymatic glycation of proteins or increased lipid peroxidation and glucose oxidation [35]. Additionally, free radicals generated in excess damage key metabolic enzymes and increase insulin resistance [9]. Furthermore, the redox imbalance modulates HIF1-α regulation and enhances the expression of the nuclear factor (NF)-κB by different, mechanisms leading to apoptosis and inflammation and finally to micro- and macro-vessel lesions [12]. In fact, NF-κB is a DNA binding protein factor involved in the transcription of numerous inflammatory molecules (e.g., cytokines, chemokines, different enzymes), with significant role in the pathogenesis of DM and its complications [11,12]. In our study, the hyperglycemic conditions induced oxidative stress and increased NF-kB and HIF1-α expressions; the process was improved by the administration of the extracts. The effect of two concentrations of both extracts on malondialdehyde was comparable and was not dependent on their phenolic acids and flavonoids contents. These results are in agreement with data from the literature and confirmed the antioxidant potential of both extracts.

It is known that NF-κB is a nuclear transcription factor involved in cellular responses to different stimuli such as free radicals, stress, cytokines, or pathogens [11,12,14]. In T1DM, its activity is not detected in β-resting cells, but exposure to proinflammatory cytokines such as IL-1 can activate it. In T2DM, NF-κB is activated by hyperglycemia and is involved in the development of insulin resistance [11]. Moreover, long-lasting hyperglycemia can induce the formation of advanced glycation end products (AGEs), which in turn can interact with specific receptors in vascular cells and generates oxidative stress, cellular dysfunctionalities, and DM complications [11,12]. Based on these data, its inhibition appears to be an important therapeutic strategy in DM and in its complications [11]. The phosphorylation of p65 NF-κB allows for the nuclear localization of the transcriptionally active complex and the transactivation of several downstream genes including inflammation, cell proliferation, and angiogenesis [36]. Therefore, the assessment of the active form p65NFkB expression may represent an indirect measure of the NF-κB activation and also of its function [14]. In our experiment, hyperglycemic medium increased the total protein level and also the activation of NF-kB in correlation with the increase of oxidative stress in HUVECs. The administration of extracts decreased the NF-kB expression in cells exposed to hyperglycemia and promoted the NF-kB activation, with an important role in cell proliferation, cell survival, and adaptive immune response [37], especially for the extract obtained from culture. The TMSE extract inhibited the NF-kB activation and protected the cells against inflammation and apoptosis. These findings are contradictory, especially since no correlation was observed with the content of total polyphenols in the extract. An explanation exists in the high phenolic acid content of the spontaneous flora or the presence of other compounds with important antioxidant activity identified in the TMSE composition. In addition, NF-kB signaling in vessel walls is complex and NF-kB pathway inhibition can be beneficial or harmful [38], depending the cell models used or the specific site for NF-kB inhibition. Generally, NF-kB is involved in the regulation of the expression of genes involved in immunity, stress responses, and inflammation and controls cell adhesion and cell proliferation. NF-kB also inhibits the activation of antiapoptotic genes such as Bcl-2 proteins, Bfl-1/A1, and BCL-XL [39] and stimulates in wall arteries the activity of nitric oxide synthase with the generation of increased amounts of nitric oxide [40]. All these results suggest the complexity of NF-kB regulation in endothelial cells and the various effects of phenolic acids or flavonoids on NF-kB expression and activation. Further studies are necessary to evaluate the behavior of the NF-kB pathway in hyperglycemia and diabetes treated with natural extracts.

Hypoxia-inducible factor 1 (HIF-1) represents the main regulator of responses to hypoxia, being formed by two subunits, HIF-1α and HIF-1β. In the case of hypoxia, of these two subunits, HIF-1α is not hydroxylated and its subunits are translocated to the nucleus, where they bind to DNA and activate the transcription of the genes responsible for hypoxia. DM not only causes hypoxia, but also compromises HIF-1 signaling, which may further be triggered by ROS. Cells become unable to respond adequately to hypoxic conditions and in this way, it increases the risk of developing complications. [13]. In our experiments, hyperglycemia enhanced the expression of HIF-1α and the tested samples helped increase it even more, therefore leading to protection against the detrimental effects of hypoxia. In fact, the relationship between glucose and HIF1-α is mutual: HIF1-α upregulates the expression of enzymes involved in glycolysis and cellular uptake while glucose influences the stability of the activation of HIF1-α [41]. HIF1-α is also involved in protective processes including angiogenesis and cell proliferation and survival by promoting the transcription of VEGF, a nitric oxide synthase and platelet-derived growth factor [42]. Therefore, the positive effect of the tested extracts on HIF1-α expression has a beneficial activity and is involved in the protection of endothelium against the detrimental effects of hypoxia or oxidative stress.

Oxidative stress can also cause alterations in the genetic material due to the toxic effects of free radicals on mitochondria. DNA alterations such as 8-oxoG, double strand breaks (DSBs), and chromosomal damage are dangerous for human cells because their persistence can initiate mutations and even carcinogenesis. Accordingly, protective mechanisms will be activated to restore the genomic integrity and prevent the mutations. In order to repair the DNA lesions, the DDR (DNA damage response) mechanism is activated. This process involves the histone H2AX, a key molecule which sends the signal for repairing proteins by phosphorylation. The formation of the γ-H_2_AX foci represents a marker for irreparable genome damages, a common process that appear in cells exposed to various genotoxic agents and in different diseases including DM [15,43]. Hyperglycemic conditions induce DNA lesions and the formation of irreparable DNA double strand breaks, a process attenuated by TMSE and TMCE administrations. These results suggest the protective role of the two tested extracts on DNA lesions, independent of the dose used or sample tested.

The link between all these parameters in hyperglycemia is represented by the oxidative stress, which activates transcription factors including NF-κB and HIF-1*α* and induces DNA lesions. The activation of these factors results in the expression of genes that control vital processes and molecules in cells, such as the cell cycle regulation or inflammatory cytokines [44]. The use of compounds with an impact on the key molecules and intracellular signaling pathways involved in the DM pathogenesis with minimal side effects and increased efficiency is welcome. The effects of the tested samples on these parameters in two different conditions, hyperglycemia and normoglycemia, bring solid arguments for their effect in diseases associated with hyperglycemia. However, further complex studies with multiple doses or exposures times are necessary to prove their clinical potency in diabetes.

## 4. Materials and Methods

### 4.1. Chemicals and Reagents

Polyphenolic compounds used as references were purchased from Merck (Darmstadt, Germany) and Fluka (Saint Louis, MO, USA). Acetonitrile for the HPLC-gradient was provided by Merck (Darmstadt, Germany) and water was purified with a Direct-Q UV system by Millipore (Darmstadt, Germany). All other chemicals used were obtained from Merck (Darmstadt, Germany). Before analysis, samples were filtered through a 0.45 µm MF-Millipore™ Membrane Filter from Merck (Darmstadt, Germany). Antibodies against NF-kB p65 (Ser536) (93H1), phospho (p)NF-kB, HIF-1α, and glyceraldehyde 3-phosphate dehydrogenase (GAPDH) and secondary peroxidase-linked antibodies were from Santa Cruz Biotechnology (Delaware Ave, Santa Cruz, CA, USA), while phosphorylated histone H2AX (pS139) (γH2AX) was from Stressgen Bioreagents Corporation (Victoria, BC, Canada). Finally, 2-thiobarbituric acid was obtained from Merck KGaA (Darmstadt, Germany) and Bradford total protein concentration assay was purchased from Bio-Rad (Hercules, CA, USA).

### 4.2. Plant Material

The aerial parts of *T*. *marschallianus* Willd. were harvested in the flowering stage in June 2019 from the spontaneous flora of North-eastern Moldavia Flora, in Cricova (voucher No. 1037), from which the extracts TMS and TMSE were obtained. The second sample was obtained from the Experimental Fields of the Botanical Garden of the Moldavian Science Academy (voucher No. 1038), from which the extracts TMC and TMCE were obtained. The identity of the species was established by Nina Ciocârlan, PhD, from the Botanical Garden of the Moldavian Science Academy, where voucher specimens are deposited in the Herbarium [14,22].

### 4.3. Preparation of Samples

Vegetal material (1 g) was individually extracted three times with 5 mL of ethanol:water (50% *v*/*v*) by sonication (Ultrasonic Cleaner, Sonica, 40 KHz, Milaono, Italy) for 1 h. The resulting mix was centrifuged (15,269× *g*) for 10 min and the supernatants were collected and stored until analysis (4 °C). The yields of the extraction were 15.3% for TMCE and 15.1% for TMSE. These initial extracts were diluted 1 to 10 with ethanol:water (70% *v*/*v*), were HPLC-DAD analyzed for the identification and quantification of polyphenolic compounds, and the total polyphenolic content was spectrophotometrically determined. Aliquots of 3 mL of sample were diluted with water to 10 mL and passed through a LiChrolut RP-18 E (40–63 mm) 500 mg/3 mL SPE column (Supelco, Sigma-Aldrich, Darmstadt, Germany). The SPE columns were washed with 10 mL of water acidified with hydrochloric acid and then eluted with 10 mL of ethyl acetate. The whole procedure was repeated 3 times and the eluted solutions were vacuum dried at a maximum of 50 °C. The dry residue was subsequently dissolved into 3 mL of ethanol:water (70% *v*/*v*) and microfiltered in order to determine the polyphenolic content by the HPLC- DAD method and the total polyphenolic content by spectrophotometric methods. Using these methods, the TMCE and TMSE samples were obtained [45].

### 4.4. HPLC-DAD Analysis of the T. marschallianus Extracts

The *T*. *marschallianus* extracts were analyzed using a Shimadzu Nexera-I HPLC apparatus. A Luna C18, silicagel-C18 150 × 4.6 mm × 3 µm column was employed for the separation of compounds. The mobile phase consisted of an acidified water–methanol gradient adjusted to pH 2.5 with 0.1% formic acid. A linear gradient was used for separation, starting with 95% water with 0.1% formic acid (A), decreasing to 75% over the next 3 min, and to 63% until the 9th minute, to 46% until the 18th minute, and finally to 5% until the 26th minute. The concentration of phase A was maintained until the 30th minute and then it was increased to 95% over the next 6 min. A 0.5 mL/minute flow rate was employed and 1 mL of sample was injected. All spectral data in the range of 190–600 nm were measured and registered. The linearity of the detector response was checked using rosmarinic acid, luteolin, apigenin, and kaempferol as references. The calibration curves data are presented in Table 3. The quantification of the polyphenolic compounds was performed on the chromatogram and recorded at 254 nm. All determinations were performed in triplicate. Data were analyzed using Excel from Microsoft Office software package [46].

### 4.5. Spectrophotometric Quantification of Total Polyphenols

The total phenolic content (TPC) was quantified using a spectrophotometric method Folin–Ciocalteu reagent as the main reagent, as stated in the European Pharmacopoeia [47,48,49]. A total of 100 µL of Folin–Ciocalteu reagent (0.2 N) was added to 10 µL of extracts and mixed with 80 µL of sodium carbonate (Na_2_CO_3_) solution (1 M). After 20 min, the absorbance of the resulting blue color was measured at 765 nm. For the quantification, a calibration curve of gallic acid was prepared with solutions in the range of 0.025–0.15 mg/mL (R^2^ = 0.9992). The results were expressed as milligrams of gallic acid equivalents (GAE) per gram of dry vegetal material.

### 4.6. Cell Culture

Commercial human umbilical vein endothelial cells (HUVECs) were purchased from the European Collection of Cell Cultures (ECACC, Porton Down, Salisbury, UK) and multiplied in RPMI medium, supplemented with 10% fetal calf serum (FCS), 50 µg/mL, of gentamicin, and 100 µg/mL of amphotericin (Biochrome AG, Berlin, Germany) in a humidified CO_2_ incubator at 37 °C. Cell cultures in the 23rd to 26th passages were used. The surfaces markers (ICAM-1, CD29, CD34, CD73, CD90, CD105) were analyzed by flow cytometry (BD FACS Canto II flow cytometer, Becton Dickinson & Company, Franklin Lakes, NJ, USA) [50].

### 4.7. Cell Viability Testing

To explore the cytotoxicity of the two *T*. *marschallianus* extracts on human umbilical vein endothelial cells (HUVECs), a CellTiter 96^®^ AQueous Non-Radioactive Cell Proliferation Assay (Promega Corporation, Madison, USA) test was employed on the human umbilical vein endothelial cells (HUVECs). The cells seeded at a density of 10^4^/well in ELISA 96-well micro titration flat bottom plaques (TPP, Switzerland) for 24 h were treated with different dilutions (between 1/80,000 and 1/20) of extract in medium ranging between 0.8 and 3200 μg GAE/mL for TMSE and between 1.175 and 4700 μg GAE/mL for TMCE, respectively, for another 24 h. The optical density values were read at an absorbance of 490 nm using an ELISA plate reader (Tecan, Männedorf, Switzerland) for MTS. All the experiments were conducted in triplicate. Untreated cultures exposed to medium were used as controls. The results were expressed as OD490.

### 4.8. Cell Lysates

HUVECs seeded at a density of 10^4^/cm^2^ in cell culture Petri dishes (TPP) were exposed for 24 h to two doses of each *T*. *marschallianus* extract—TMCE: 0.94 μg GAE/mL (sample TMCE1) and 0.094 μg GAE/mL (sample TMCE2) and TMSE: 0.66 μg GAE/mL (sample TMSE1) and 0.066 μg GAE/mL (sample TMSE2)—either in medium with 200 mmol/L glucose (hyperglycemic stress) or in medium with 137 mmol/L glucose (normoglycemic conditions). Then, the cells were washed three times and incubated for another 24 h in standard conditions. After that, the cells were collected by scraping and treated with a lyses buffer containing IGEPAL Nonidet 1% (Sigma-Aldrich, Darmstadt, Germany) and 1% protease inhibitor complex (Sigma) in PBS for 1 h on ice. The protein content was determined by the Bradford method (Bio-Rad, Hercules, California, USA) according to the manufacturer’s specifications. All the experiments were performed in triplicate. For all assays the lysates were corrected by total protein concentration. The cell lysates were used for MDA assessment and Western blot analysis.

### 4.9. Oxidative Stress Assessment

Lipid peroxides were determined by using the fluorimetric method with 2-thiobarbituric acid (TBA) as described by Conti et al. [51] and were expressed as malondialdehyde (MDA) (nmol/mg protein).

### 4.10. Transcription Factors Expressions and DNA Lesions Evaluation

The expression of transcription factor NF-kB and its phosphorylated form (pNF-kB), HIF1-α, and DNA lesions (γH2AX) were evaluated by Western blot analysis. Lysates (20 µg protein/lane) were separated by electrophoresis on SDS PAGE gels (Bio-Rad) and transferred to polyvinylidene difluoride membranes (Bio-Rad, USA). Blots were blocked for 1 h and then incubated with antibodies against NF-kB, pNF-kB, HIF1-α, γH2AX, GAPDH, and the corresponding secondary peroxidase-coupled antibodies (Santa Cruz Biotechnology). The proteins were visualized and detected using Supersignal West Femto Chemiluminiscent substrate (Thermo Fisher Scientific, Rockford, IL, USA) and quantified using Quantity One analysis software (Bio-Rad). GAPDH was used as the protein loading control [52].

### 4.11. Statistical Analysis

The statistical significance of the difference between the treated and control groups was evaluated with the two-way ANOVA followed by a Bonferroni post-hoc test using GraphPad Prism version 5.00 for Windows (GraphPad Software, San Diego, CA, USA, www.graphpad.com, accessed on 1 October 2021). A *p* value < 0.05 was considered statistically significant.

## 5. Conclusions

Polyphenolic-enriched extracts obtained from *T*. *marschallianus* (Lamiaceae) harvested from culture and from spontaneous flora contain significant amounts of rosmarinic acid, luteolin, kaempferol, and apigenin. Their biological effects were tested on HUVECs exposed to normoglycemia and hyperglycemia and showed a significant reduction of oxidative stress and of the γH2AX formation and significantly improved the expression of the HIF1-α. Moreover, they reduced the expression of the total pNF-kB and its activation. All these extracts clearly showed a protective role on endothelial cells and helped to bring further arguments that sustain the use of *T*. *marschallianus* in the protection against the effects of hyperglycemia in DM and its complications.

## Figures and Tables

**Table 1:** Results obtained for the quantification of TPA, TFC, and TPC by HPLC-DAD * and spectrophotometrical ** methods.

Sample	TPA (mg CAE/g) *	TFC (mg RE/g) *	TPC (mg GAE/g) **
TMC	3.16 ± 0.08	2.49 ± 0.05	5.72 ± 0.12
TMCE	3.19 ± 0.02	6.78 ± 0.05	10.34 ± 0.15
TMS	3.32 ± 0.01	1.45 ± 0.04	5.39 ± 0.10
TMSE	4.02 ± 0.07	2.83 ± 0.01	7.26 ± 0.11

Note: Values represent the mean ± standard deviations of three independent measurements.

**Table 2:** Results obtained for the HPLC quantification of polyphenolic compounds.

Sample	Rosmarinic Acid (mg/g)	Luteolin (mg/g)	Kaempferol (mg/g)	Apigenin (mg/g)
TMC	0.18 ± 0.02	2.04 ± 0.10	1.85 ± 0.01	4.97 ± 0.02
TMCE	0.31 ± 0.11	17.71 ± 0.52	7.39 ± 0.02	65.67 ± 0.09
TMS	0.18 ± 0.54	4.17 ± 0.11	2.75 ± 0.01	5.50 ± 0.02
TMSE	0.24 ± 0.32	5.69 ± 0.25	5.69 ± 0.02	14.63 ± 0.04

Note: Values represent the mean ± standard deviations of three independent measurements.

**Table 3:** The data obtained for the reference calibration curves.

Standard	Concentration Range (mg/mL)	R^2^	Detection Limit (mg/mL)	Quantification Limit (mg/mL)
Rosmarinic acid	56–240	0.9946	3.5	7.1
Luteolin	40–300	0.9945	27.2	54.4
Kaempferol	40–300	0.9799	40.6	81.3
Apigenin	48–360	0.9990	15.6	26.1
Rutoside	300–500	0.9984	2.21	4.42
Caffeic acid	300–500	0.9994	124	186

## Data Availability

Data sharing not applicable.
